# Targeting the Gut–Kidney Axis: Modulation of Gut Microbiota by Traditional Chinese Medicine for Chronic Kidney Disease Management

**DOI:** 10.3390/toxins17120599

**Published:** 2025-12-15

**Authors:** Yijing Xin, Libin Pan

**Affiliations:** 1 Department of Clinical Pharmacy, The First Affiliated Hospital, Zhejiang University School of Medicine, Hangzhou 310003, China; yijingxin@zju.edu.cn; 2 Department of Pharmacy, Zhejiang Cancer Hospital, Hangzhou Institute of Medicine (HIM), Chinese Academy of Sciences, Hangzhou 310022, China

**Keywords:** chronic kidney disease, microbial metabolites, gut microbiota, traditional Chinese medicine, gut–kidney axis

## Abstract

The interaction between gut microbiota dysbiosis and CKD progression via the “gut–kidney axis” is increasingly recognized. Gut-derived uremic toxins (e.g., indoxyl sulfate and p-cresyl sulfate) accumulate systemically, while beneficial metabolites like short-chain fatty acids (SCFAs) decrease, contributing to inflammation, oxidative stress, and kidney fibrosis. Traditional Chinese Medicine (TCM), including complex formulae, single herbs, and active ingredients, has long been used to manage CKD. Emerging evidence—primarily from animal studies—highlights its potential to alleviate the disease by modulating the gut microbiota. This review summarizes how TCM interventions re-establish gut microbial symbiosis by regulating microbial composition, reducing toxin load, and reinforcing intestinal barrier integrity, thereby ameliorating systemic inflammation and protecting kidney function. Targeting the gut microbiota represents a promising therapeutic frontier for CKD, and TCM offers a rich resource for developing novel microbiota-modulating strategies. However, future research must focus on validating molecular mechanisms, standardizing TCM preparations, and conducting rigorous clinical trials to facilitate clinical translation.

## 1. Introduction

Chronic kidney disease (CKD) is a major global public health problem, consuming substantial financial and social resources and accompanied by a significant decline in patients’ quality of life and life expectancy [1]. In 2022, the global number of CKD patients was approximately 850 million [2]. Moreover, the disease represents a significant mortality burden and is projected to become the fifth leading cause of death globally by 2040 [3]. The prevalence of CKD gradually increases with age, with its primary risks lying in diabetes, hypertension, and obesity [4], as well as increased susceptibility due to variations in certain genes [5].

In recent years, with the deepening of gut microbiota research, the association between gut microbiota and kidney disease and function has been progressively uncovered. The “gut–kidney axis” concept was first proposed in 2011 to explain how changes in gut microbial ecology influence the course of CKD by modulating metabolites [6]. The physiological structure of the gut supports its unique immune and metabolic functions. The intestine harbors a vast number and diversity of metabolic enzymes, serves as the site for the digestion of nutrients and metabolites, and is the largest source of uremic toxins in the body [7]. Consequently, the role of gut microbiota in the pathogenesis, disease management, and prognosis of CKD has garnered widespread attention. Recent studies have found that, in CKD, an increase in pathobionts alongside a decrease in commensal taxa with beneficial metabolic profiles leads to dysbiosis, directly damaging the intestinal barrier. This results in bacterial translocation and the accumulation of gut-derived uremic toxins in the blood, activating inflammation, oxidative stress, and kidney fibrosis, thereby exacerbating kidney damage [8]. Furthermore, excess metabolic waste that cannot be adequately excreted by the kidneys re-enters the intestinal lumen, further aggravating intestinal microbiota disturbance and creating a vicious cycle (reverse causality), which is further modulated by host factors such as diet and systemic inflammation [9]. While uremic toxin pathways have been detailed in recent reviews [10], the potential of TCM to intervene in this cycle warrants specific attention. Notably, emerging evidence from both preclinical and clinical studies suggests that fecal microbiota transplantation (FMT) can attenuate disease progression and stabilize kidney function by reversing gut microbiota dysbiosis [11,12,13].

Traditional Chinese medicine (TCM) has been used for many years in China for the treatment of and intervention in CKD, demonstrating significant therapeutic effects and functions [14]. It is noteworthy that growing evidence suggests that the efficacy of TCM is closely related to its ability to modulate the structure and function of the gut microbiota and influence microbial metabolites [15]. Recent mechanistic studies utilizing multi-omics and microbial–metabolite network analysis have begun to decode these complex drug–host–microbiota interactions [16,17,18]. However, challenges regarding the safety and standardization of herbal products remain critical topics for discussion. Furthermore, it is particularly crucial to deeply explore the mechanisms of action of TCM (including formulae and monomeric compounds) based on the “gut–kidney axis”. This narrative review synthesizes the complex relationships among TCM (formulae/monomers), gut microbiota, and chronic kidney disease (CKD) from the perspective of gut microbiota. For this review, relevant studies were retrieved from four core databases (PubMed, Embase, Web of Science, and China National Knowledge Infrastructure [CNKI]) with a search range from January 2010 to December 2024. The key search terms included “chronic kidney disease”, “gut microbiota”, “traditional Chinese medicine”, “herbal formula”, “active monomer”, and “gut–kidney axis”. The literature selection was based on the following criteria: (1) original research (in vitro, in vivo, or clinical studies) focusing on TCM-mediated gut microbiota modulation in CKD; (2) studies providing clear data on gut microbiota composition, microbial metabolites, or intestinal barrier function; (3) publications in peer-reviewed English or Chinese journals. Studies were excluded if they were reviews, meta-analyses, case reports, conference abstracts, studies lacking direct links between TCM and gut microbiota–CKD crosstalk, or duplicate/incomplete data. This review aims to provide new theoretical foundations and strategic insights for CKD intervention.

## 2. Mechanisms of Chronic Kidney Disease Based on Gut Microbiota

### 2.1. Production and Activation Pathways of Uremic Toxins

Uremic toxins refer to specific retention solutes that accumulate in the blood when kidney excretory function is impaired and have been demonstrated to exert deleterious biological effects, and the gut microbiota is a significant source of these toxins. The accumulation of uremic toxins leads to the progression of CKD and various cardiovascular complications, such as endothelial activation/dysfunction, atherosclerosis, cardiomyocyte apoptosis, cardiac fibrosis, and vascular and valvular calcification, consequently causing hypertension, arrhythmia, myocardial infarction, and cardiomyopathy [19]. Studies show that blood metabolites are primarily mediated by diet and gut microbiota: specifically, diet explains the interindividual variations in blood concentrations of 335 metabolites, while the gut microbiota accounts for those of 182 metabolites [20]. Notably, a recent large-scale clinical study characterized 58 protein-bound uremic toxins in the blood and detected significant associations between 16 uremic toxins and 97 microbes [21]. As research deepens, various gut microbiota-derived uremic toxins have been gradually characterized, including p-cresyl sulfate (pCS), indoxyl sulfate (IS), indole-3-acetic acid, and trimethylamine, among others. The following sections review the production pathways and toxic mechanisms of these key uremic toxins (i.e., the ones highlighted above).

#### 2.1.1. p-Cresyl Sulfate (pCS)

pCS is primarily produced by specific gut bacteria via the tyrosine pathway. For instance, *Clostridioides difficile* ferments tyrosine to produce p-cresol via the intermediate p-hydroxyphenylacetate (p-HPA). The p-HPA decarboxylase, encoded by the *hpdB* operon, decarboxylates p-HPA to produce p-cresol. Once produced, p-cresol is sulfated to form pCS, a process occurring mainly in the colonic mucosa and liver [22,23]. Elevated pCS levels are associated with an increased risk of cardiovascular disease, particularly in individuals with impaired kidney function. The presence of pCS in the blood can promote a pro-thrombotic phenotype, leading to CKD [24,25]. In CKD patients, the accumulation of pCS is associated with various complications, including immune dysfunction and oxidative stress, which exacerbate CKD progression [26].

#### 2.1.2. Indoxyl Sulfate (IS)

IS production in healthy individuals ranges approximately from 10 to 130 mg per day [27]. IS is metabolized from indole, which is converted from dietary tryptophan by gut microbial tryptophanase [28,29]. Indole production involves specific gut microbiota and enzyme systems; for example, *Escherichia coli* and *Bacteroides fragilis* can metabolize tryptophan into indole via tryptophanase (EC 4.1.99.1) [30]. Indole is then absorbed in the liver and converted to IS by CYP2E1 and SULT1A1 enzymes [29]. A recent study using an oral tryptophan challenge test indicated that blood IS levels are primarily regulated by variations in gut microbiota rather than by the abundance of host CYP2E1 and SULT1A1.

IS affects the progression of CKD through multiple mechanisms: IS induces oxidative stress and inflammation, increases reactive oxygen species (ROS), and reduces antioxidant responses, affecting various signaling pathways such as nuclear factor kappa-light-chain-enhancer of activated B cells (NF-κB), tumor protein p53 (p53), signal transducer and activator of transcription 3 (STAT3), transforming growth factor-beta (TGF-β) and Smad2/3 [31,32,33]. IS is a significant cardiovascular risk factor in CKD, activating macrophages via the OATP2B1 transporter and Notch signaling pathway, promoting vascular inflammation and leading to atherosclerosis and other cardiovascular diseases [34,35]. IS directly induces kidney tubular cell apoptosis and necrosis, exacerbating tubulointerstitial injury and kidney fibrosis, and upregulates TGF-β1 and other pro-inflammatory markers, further promoting CKD progression [33,36]. IS can also affect the intestinal system by inducing oxidative stress in intestinal epithelial cells, impairing cell migration, and disrupting the intestinal barrier [32].

#### 2.1.3. Trimethylamine/Trimethylamine N-Oxide (TMA/TMAO)

Trimethylamine (TMA) is a metabolite generated by the gut microbial metabolism of dietary choline and carnitine. TMAO formation is a multi-step process: first, gut microbes convert dietary precursors (e.g., choline, carnitine, betaine) into trimethylamine (TMA), which is then oxidized in the liver to TMAO by flavin monooxygenases (FMOs) [37,38,39]. TMAO is primarily excreted by the kidneys; hence, its plasma concentration is significantly increased in CKD patients [40,41]. TMAO plays an important role in the progression of CKD through the following mechanisms: TMAO exacerbates kidney injury by inducing oxidative stress [40,42]; TMAO is a potent pro-inflammatory factor, capable of activating the NLRP3 inflammasome and promoting the expression of inflammatory cytokines such as TNF-α, IL-6, and IL-1β [42,43,44]; TMAO further impairs kidney function by inducing endoplasmic reticulum stress; TMAO promotes kidney fibrosis by inducing ferroptosis in kidney tubular epithelial cells and the secretion of fibrotic factors [45,46]. High levels of TMAO are significantly associated with cardiovascular events and all-cause mortality in CKD patients [40,47]; furthermore, TMAO is associated with the progression to CKD following acute kidney injury (AKI), especially after kidney ischemia–reperfusion injury [48].

The production of TMA in the gut relies on specific metabolic enzymes responsible for converting dietary precursors into TMA. Key core enzymes include choline TMA-lyase (CutC) [49], which converts choline to TMA; carnitine monooxygenase (CntA) [50], responsible for converting L-carnitine to TMA; and γ-butyrobetaine reductase, involved in converting γ-butyrobetaine to TMA. Key bacteria involved in TMA production include *Escherichia coli*, *Enterobacter*, *Proteus*, and certain *Clostridium* species [51]. These bacteria, through their unique metabolic pathways, promote TMA production and play important roles in the gut’s microecology.

#### 2.1.4. Hippuric Acid (HA)

Hippuric acid (HA) is an abundant metabolite in mammalian urine, primarily produced via host–gut microbiota co-metabolic pathways [52], often derived from the gut microbial catabolism of dietary polyphenols [53], or as a metabolite resulting from the hepatic glycine conjugation of benzoic acid (BA) or the metabolism of phenylalanine by gut bacteria [54]. Recent literature also reports a novel mechanism where gut bacteria (e.g., *Clostridium sporogenes*) reduce phenylalanine to phenylpropionic acid (PPA) via its fldC gene cluster; the host absorbs PPA, which then undergoes β-oxidation via medium-chain acyl-CoA dehydrogenase (MCAD) to produce benzoic acid, ultimately conjugating with glycine to form hippuric acid [52]. HA is a protein-bound uremic toxin that accumulates in CKD patients and is associated with disease progression and kidney fibrosis. HA levels positively correlate with CKD progression, suggesting that higher HA levels may exacerbate the condition [55,56]; HA promotes kidney fibrosis by disrupting redox homeostasis and increasing oxidative stress, a key pathological process in CKD [55]; HA increases the production of ROS, which are detrimental to kidney cells; HA reduces nuclear factor erythroid 2-related factor 2 (NRF2) levels via NRF2 ubiquitination, thereby disrupting antioxidant defenses. This leads to ROS accumulation and subsequent fibrotic responses [55]. HA levels are associated with left ventricular hypertrophy (LVH) in hemodialysis patients, suggesting their potential as a biomarker for cardiovascular complications in CKD. HA, along with other uremic toxins like IS and pCS, is associated with coronary atherosclerosis, contributing to cardiovascular risk in CKD patients [57]; HA levels independently correlate with several hemodialysis quality indicators, including blood pressure, β2-microglobulin, and creatinine levels, suggesting that HA could serve as a marker for assessing the effectiveness of hemodialysis treatment [51]; HA levels are associated with tubular injury markers, such as kidney injury molecule 1 (Kim-1) [58]. CKD patients often experience metabolic acidosis due to impaired kidney function. HA, as a uremic toxin, contributes to this by accumulating in the plasma and affecting acid–base balance [59].

#### 2.1.5. Urea

Urea is the primary end product of protein metabolism. Its blood levels are traditionally evaluated via blood urea nitrogen (BUN), a clinical marker of kidney function and dialysis efficiency [60]. Historically, urea was considered biologically inert [61]. However, recent experimental data show that urea exhibits multi-organ toxicity at concentrations typical of CKD. Urea induces endothelial dysfunction and apoptosis in vascular smooth muscle cells, leading to cardiovascular complications [62]. High urea levels disrupt the intestinal epithelial barrier, leading to bacterial toxin translocation into the bloodstream and systemic inflammation [63]. Urea catabolism produces isocyanic acid, which carbamylates proteins, altering their structure and function—a process linked to kidney fibrosis, atherosclerosis, and anemia [61]. The urea level is an important clinical indicator in CKD. Elevated urea levels (corresponding to increased BUN in clinical settings) are associated with higher inflammatory markers in CKD patients, such as the neutrophil–lymphocyte ratio (NLR) [64]. High BUN levels are inversely related to hemoglobin levels, increasing the risk of anemia in CKD patients [65]. The BUN-to-Albumin Ratio (BAR) is a significant predictor of 28-day mortality in ICU patients with CKD [66]. Furthermore, CKD leads to elevated urea levels (clinically reflected by higher BUN), which in turn alter the composition of the gut microbiota. This dysbiosis leads to the production of uremic toxins, such as IS and pCS, thereby exacerbating kidney damage and systemic inflammation [67].

Urea is an important nitrogen source for gut microbiota; therefore, increased intestinal urea levels due to compensatory intestinal excretion significantly regulate the composition and function of the gut microbiota [68]. Urease is the main enzyme cluster for urea decomposition by gut microbiota, mediating ammonia production. Studies have reported that various gut microbes are involved in urea-mediated CKD progression. For example, Alistipes indistinctus and Alistipes putredinis promote CKD progression through interactions with immune cells [69]; Bacteroides increase in CKD and are associated with higher levels of uremic toxins and systemic inflammation [70].

#### 2.1.6. Integrative View of Microbial Biosynthesis Pathways

**Figure 1:**
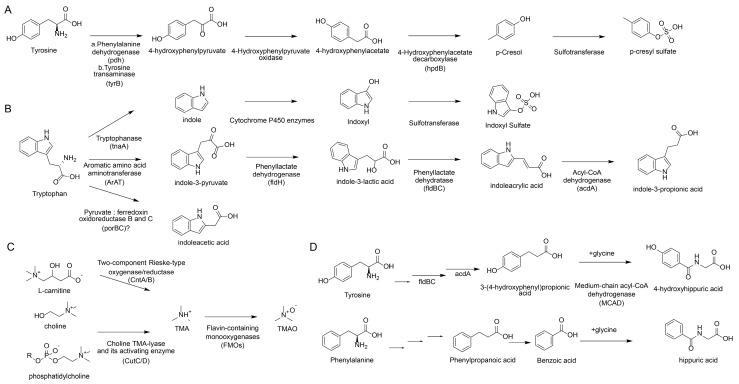
Metabolic pathways of common uremic toxins and the main mediating gut microbiota enzymes. (**A**) Generation pathway of pCS. (**B**) Generation pathway of IS. (**C**) Generation pathway of TMA/TMAO. (**D**) Generation pathway of hippuric acid.

Collectively, the production of the aforementioned uremic toxins is not stochastic but is strictly orchestrated by specific bacterial gene clusters acting on distinct dietary substrates, as illustrated in Figure 1. From a unified biosynthetic perspective, these pathways can be categorized into two main streams. The first stream involves amino acid metabolism. The generation of phenolic toxins (pCS) and indolic toxins (IS) relies on bacterial enzymes that process tyrosine and tryptophan, respectively. Key enzymes include tyrosine transaminase (tyrB) and 4-hydroxyphenylacetate decarboxylase (hpdB) found in *Clostridium* for p-cresol synthesis, and tryptophanase (tnaA) in *Escherichia coli* for indole production [71]. Notably, a competitive pathway involves phenyllactate dehydratase (fldBC) in *Clostridium sporogenes* [72], which diverts tryptophan towards the beneficial metabolite IPA. The second stream targets quaternary amines (choline/carnitine). This specific pathway for TMAO precursors is driven by the choline TMA-lyase (CutC) and its activating enzyme (CutD), predominantly expressed in *Proteus mirabilis* [73] and *Klebsiella pneumoniae* [74]. Understanding these upstream “enzymatic switches”—rather than just the downstream toxic effects—provides precise molecular targets. This highlights that the therapeutic potential of TCM may lie in its ability to inhibit these specific bacterial enzymes (e.g., tnaA, CutC) or suppress the specific taxa harboring them, thereby cutting off the source of uremic toxicity.

### 2.2. Beneficial Factors Derived from Gut Microbiota

#### Short-Chain Fatty Acids (SCFAs)

Short-chain fatty acids (SCFAs) are a class of fatty acids with no more than six carbon atoms, primarily produced in the gut through microbial fermentation of dietary fiber. SCFAs include acetate, propionate, and butyrate [75]. SCFAs play a crucial role in maintaining gut health by modulating metabolism, supporting gut barrier function, and reducing inflammation [76]. They are the primary energy source for colonocytes (cells lining the colon) [77]. SCFAs have significant immunomodulatory functions, affecting both local and systemic immune responses. They are involved in regulating glucose and lipid metabolism, thereby influencing conditions like obesity, diabetes, and cardiovascular disease [78]. Compared to in healthy individuals, SCFA levels in the blood are significantly lower in CKD patients, and this reduction is associated with the gut microbiota dysbiosis common in CKD [79]. Specific SCFAs, such as butyrate, are significantly reduced in CKD patients, and this reduction correlates with disease severity [80]. SCFAs, particularly butyrate, have shown potential renoprotective effects. Butyrate supplementation in CKD models has been found to alleviate kidney fibrosis and reduce inflammation by modulating pathways such as NLRP3-mediated pyroptosis and the STING/NF-κB/p65 pathway [81]. SCFAs can also modulate inflammation and oxidative stress, key factors in CKD progression. SCFAs exert their beneficial effects through various mechanisms, including the following: SCFAs reduce pro-inflammatory parameters and oxidative stress, which are crucial in CKD pathogenesis [82]; histone deacetylase inhibition, leading to epigenetic modifications that can protect against kidney injury [83,84]; modulation of the gut microbiota. Dietary interventions that increase SCFA-producing bacteria can improve gut health and potentially slow CKD progression [85].

**Figure 2:**
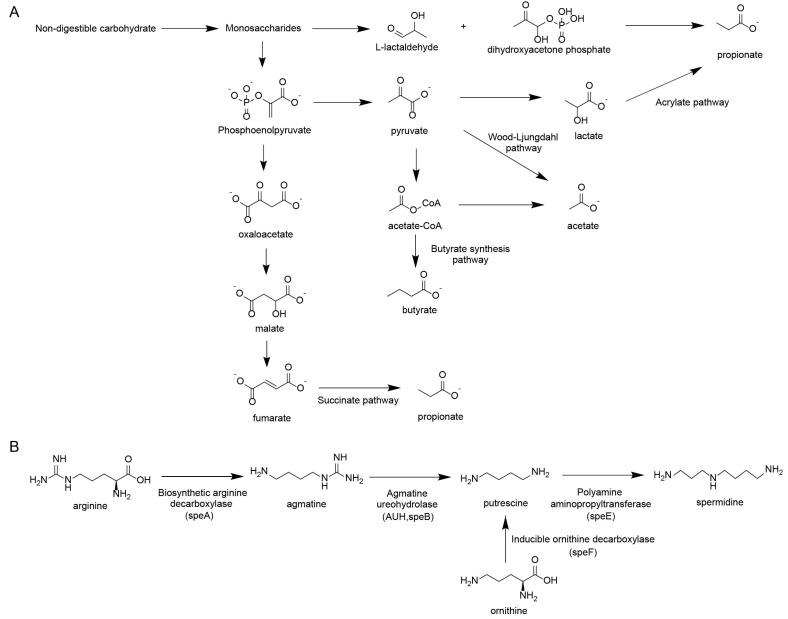
Other key metabolites derived from gut microbiota. (**A**) Generation pathway of short-chain fatty acids. (**B**) Generation pathway of polyamines.

Regarding carbohydrate fermentation (Figure 2A), the production of propionate and butyrate follows distinct enzymatic routes. Propionate is synthesized primarily via the succinate pathway (converting phosphoenolpyruvate to succinate) or the acrylate pathway (converting lactate to propionate). Butyrate synthesis largely relies on the conversion of acetyl-CoA through the butyrate synthesis pathway.

### 2.3. Other Important Gut Microbiota Metabolites

#### Polyamines

Polyamines, such as putrescine, spermidine, and spermine, play vital roles in cellular functions, including cell growth, differentiation, and apoptosis. These compounds are synthesized by both mammalian cells and the gut microbiota, and their production is significantly influenced by the composition of the gut microbiome and the host’s diet [86]. The gut microbiota is an important source of polyamines, with specific bacteria, such as *Lactobacillus murinus* and *Bacteroides* spp., being major contributors [87]. Polyamines offer several benefits in CKD, such as reducing kidney fibrosis, a key factor in CKD progression. Studies show decreased spermine and increased spermidine levels in CKD, suggesting that altered polyamine metabolism contributes to CKD progression [88]; spermidine and spermine possess antioxidant and anti-inflammatory properties. These properties help reduce oxidative stress and inflammation, important factors contributing to kidney damage in CKD [89]. In terms of improving autophagy and reducing senescence, supplementation with exogenous spermine or genetic deficiency of spermine oxidase (SMOX) has been shown to improve autophagy, reduce cellular senescence, and attenuate fibrosis in a mouse model of CKD [88]. This indicates a protective role for spermine in maintaining kidney function. Furthermore, serum levels of polyamines like putrescine, spermidine, and spermine can serve as potential biomarkers for CKD progression [90]. Higher levels of spermidine and spermine are associated with better kidney function and a lower risk of cardiovascular events and mortality [91].

However, it is crucial to address the apparent “duality” of polyamines in CKD. While polyamines are classically categorized as uremic toxins, this toxicity is primarily driven by the systemic accumulation of the precursor putrescine and the toxic byproducts (e.g., acrolein and H_2_O_2_) generated by enhanced catabolism via enzymes like SAT1 and SMOX. In contrast [92], the higher-order polyamines, spermidine and spermine, are often depleted in CKD patients [93]. This depletion represents a loss of protective mechanisms, as these specific polyamines are essential for maintaining mitochondrial homeostasis and inducing autophagy [93]. Therefore, the therapeutic strategy should focus on correcting the deficiency of protective spermidine/spermine while mitigating the oxidative stress caused by their excessive catabolism.

Regarding amino acid metabolism (Figure 2B), the synthesis of polyamines is governed by precise bacterial gene clusters acting on arginine and ornithine. Key enzymatic steps include the conversion of arginine to agmatine by biosynthetic arginine decarboxylase (speA), and subsequently to putrescine by agmatine ureohydrolase (speB). Alternatively, ornithine is directly decarboxylated to putrescine by ornithine decarboxylase (speF). Putrescine is then converted to spermidine by spermidine synthase (speE).

## 3. The Pivotal Role of TCM Against CKD-Related Metabolic Disorders

**Figure 3:**
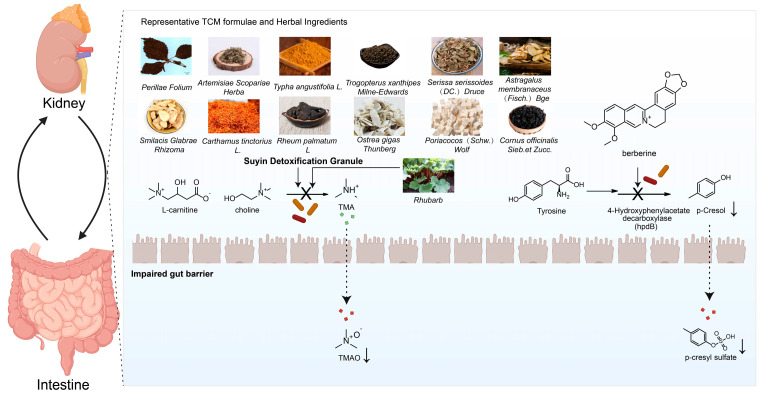
Schematic diagram illustrating the interactions between representative TCM formulae and herbal ingredients and gut microbiota metabolites.

With its long history of use, TCM holds significant value in managing CKD. TCM formulae and active compounds demonstrate therapeutic potential by targeting multiple mechanisms, including the inhibition of fibrosis and regulation of mitochondrial function. Notably, their modulation of the “gut–kidney axis” reduces uremic toxins and suppresses the growth of pathogenic bacteria, highlighting a promising therapeutic approach. A substantial body of basic research has demonstrated that herbal formulae, single herbs, and their active ingredients can ameliorate metabolic disorders, reduce kidney injury, and delay the progression of CKD by modulating the structure, composition, and metabolic function of the gut microbiota [94]. This section will elaborate on the underlying mechanisms from these three perspectives, illustrating the interplay between representative TCM formulae and herbal ingredients and gut microbiota metabolites (see Figure 3 for details).

### 3.1. Herbal Formulae

Herbal formulae, with their multi-component and multi-target characteristics, demonstrate comprehensive advantages in correcting gut microbiota dysbiosis associated with CKD (Table 1). For instance, Yi-Shen-Hua-Shi formula was shown in CKD patients to significantly increase the abundance of beneficial bacteria (e.g., *Faecalibacterium*, *Lachnospiraceae*) and reduce pathogenic bacteria (e.g., *Eggerthella*), which correlated with a significant decrease in 24 h proteinuria [95]. Jian-Pi-Yi-Shen (JPYS) decoction, in 5/6 nephrectomized rats, not only restored the levels of phyla like Firmicutes but also enriched butyrate-producing genera (e.g., *Coprococcus*, *Phascolarctobacterium*), concurrently reducing BUN and urinary albumin (U-ALB) levels [96].

Similarly, the combination of *Astragalus membranaceus* and *Salvia miltiorrhiza* reversed the Firmicutes/Bacteroidetes ratio (F/B ratio), and increased the abundance of probiotics like butyrate-producing *Akkermansia* and lactate-producing *Lactobacillus*, thereby lowering uremic toxins, improving inflammation and oxidative stress, and protecting kidney function [97]. Research on Suyin Detoxification Granule revealed that it counteracts dysbiosis by reversing the F/B shift and suppressing genera like *Muribaculaceae*, leading to reduced circulating TMAO levels and inhibition of TMAO-induced kidney tubular ferroptosis and fibrosis, thus preventing CKD progression [98]. Other formulae, such as Fu-Zheng-Hua-Yu-Jiang-Zhu-Tong-Luo-Fang and Yiqi–Huoxue–Jiangzhuo formula, have also been confirmed to ameliorate kidney fibrosis and improve kidney function by regulating specific gut microbes. Relevant studies are summarized in Table 1.

**Table 1:** Basic studies on the potential mechanism of herbal formulae against CKD.

Formula Composition	Subject	Subject Size	Gut Microbiota Study Method	Formula Composition	Effect on GM	Relief or Treatment of Symptoms	Reference
Yi-Shen-Hua-Shi	CKD patients	Renin–angiotensin–aldosterone system (RAAS) inhibitor plus YSHS (*n *= 56) or RAAS inhibitor (*n* = 47)	16S rRNA sequencing	Ginseng Redix et Rhizoma, Astragali Radix, Atractylodis Macrocephalae Rhizoma, Poria, Alismatis Rhizoma, Pinelliae Rhizoma Praeparatum Cum Alumine, Notopterygii Rhizoma et Radix, Angelicae Pubescentis Radix, Saposhnikoviae Radix, Bupleuri Radix, Copidis Rhizoma, Paeoniae Radix Alba, Citri Reticulatae Pericarpium, Glycyrrhizae Radix et Rhizoma praeparata Cum Melle, Zingiberis Rhizoma Recens and Jujubae Fructus.	Increased the relative abundance of bacteria that have beneficial effects on the body, such as *Faecalibacterium*, *Lachnospiraceae*, *Lachnoclostridium*, and *Sutterella*; decreased pathogenic bacteria such as the *Eggerthella* and *Clostridium innocuum* group	Patients in the intervention group had a significantly higher decrease in 24 h proteinuria	[95]
Jian-Pi-Yi-Shen decoction	5/6 nephrectomized rats	*n* = 10	16S rRNA sequencing	Astragali Radix, Atractylodis Macrocephalae Rhizoma, Dioscoreae Rhizoma, Cistanches Herba, Amomi Fructus Rotundus, Salviae Miltiorrhizae Radix et Rhizoma, Rhei Radix et Rhizoma, and Glycyrrhizae Radix et Rhizoma Praeparata cum Melle	Restored the levels of Firmicutes, Actinobacteria, and Proteobacteria. Enriched the butyrate-producing genera *Coprococcus*, *Phascolarctobacterium*, *Parasutterella*, and *Clostridium_XlVb*	Reduced BUN level and U-ALB level; restored the blood reticulocyte and serum calcium levels	[96]
Jian-Pi-Yi-Shen formula	Adenine-induced CKD rats	*n* = 5	Metagenomics	Astragali Radix, Atractylodis Macrocephalae Rhizoma, Dioscoreae Rhizoma, Cistanches Herba, Amomi Fructus Rotundus, Salviae Miltiorrhizae Radix et Rhizoma, Rhei Radix et Rhizoma, and Glycyrrhizae Radix et Rhizoma Praeparata cum Melle	Decreased Aitchison distances, reflecting more substantial gut microbiota changes	Reduced serum creatinine levels, particularly during the recovery phase following adenine withdrawal	[99]
The combination of Astragalus membranaceus and Salvia miltiorrhiza	Cyclosporin A-induced rats	*n* = 8	16S rDNA sequencing	Astragalus membranaceus and Salvia miltiorrhiza	Reversed the ratio of Firmicutes to Bacteroidetes. Increased the abundance of probiotics producing butyric acid (*Akkermansia*) and lactic acid (*Lactobacillus*)	Reduced the level of urinary toxins, improved the state of inflammation and oxidative stress, and then protected kidney function; showed an amelioration of pathological injuries to varying degrees, especially in the diminished collagen volume fraction	[97]
You-gui pill	Hydrocortisone-induced kidney-Yang deficiency syndrome rats	*n* = 10	16S rRNA sequencing	Radix Rehmanniae Praeparata (SDH), Radix Aconiti Lateralis Prreparata (FZ), Cinnamomi cortex (RG), Rhizoma Dioscoreae (SY), Fructus Corni (SZY), Semen Cuscutae (TSZ), Fructus Lycii (GQ), Cervi cornus colla (LJJ), Eucommiae cortex (DZ), and Radix Angelicae Sinensis (DG)	Increased Firmicutes level but decreased Bacteroidetes level. Regulated four kinds of microbes (i.e., *Ruminiclostridium_9*, *Ruminococcaceae_UCG-007*, *Ruminococcaceae_UCG-010*, and *uncultured_bacterium_f_Bacteroidales_S24-7_group*)	Ameliorated hypothalamic–pituitary–target gland axis dysfunction and pathological damage in rats with kidney-Yang deficiency syndrome	[100]
Suyin Detoxification Granule	Adenine-induced CKD rats with 1% high-choline diet	*n* = 6	16S rRNA sequencing	Perilla frutescens (L.) Britton [Zisuye] (330 g), Artemisia capillaris Thunb. [Yinchen] (165 g), Serissa japonica (Thunb.) Thunb. [Liuyuexue] (220 g), Smilax glabra Roxb. [Tufuling] (330 g), Carthamus tinctorius L. [Honghua] (110 g), Typha angustifolia L. [Puhuang] (110 g), Rheum officinale Baill. [Dahuang] (88 g), Poria cocos (Schw.) Wolf [Fuling] (110 g), Astragalus membranaceus (Fisch.) Bunge [Huangqi] (330 g), Cornus officinalis Siebold & Zucc. [Shanzhuyu] (110 g), Ostrea gigas Thunberg [Muli], Faeces Trogopterori [Wulingzhi] (110 g)	Counteracted the dysbiosis by reversing the Firmicutes/Bacteroidota shift, suppressing genera like *Muribaculaceae*, *F082*, and *Bacteroides*, and prevented the reduction in *Lactobacillus*	Reduced circulating TMAO levels and inhibited TMAO-induced kidney tubular ferroptosis, profibrotic factor secretion, and kidney fibrosis to prevent CKD progression	[98]
Fu-Zheng-Hua-Yu-Jiang-Zhu-Tong-Luo-Fang prescription	Unilateral ureteral obstruction (UUO) rat model	*n* = 12	16S rRNA sequencing	raw astragalus, Rehmannia glutinosa, Salvia, safflower, wine leeches, soil beetle, wine Scutellaria, wine rhubarb, and raw licorice	Counteracted gut microbiota dysbiosis. Reduced overall microbial diversity, decreased the abundance of 10 elevated genera (e.g., *g_Monoglobus*, etc.), and reversed the enrichment of 4 UUO—increased pathogenic bacteria	Improved kidney function by reducing serum creatinine and BUN levels, alleviated kidney tissue damage, and markedly reversed fibrosis	[101]
Yiqi–Huoxue–Jiangzhuo formula	5/6 nephrectomized mice	*n* = 6	16S rRNA sequencing	Astragali Radix, Angelicae Sinensis Radix, Salviae Miltiorrhizae Radix Et Rhizoma, Rhei Radix Et Rhizoma, and Herba Hedyoti Diffusae	Countered 5/6 Nx-induced rise in Bacteroidetes and fall in Firmicutes. Restored Ruminococcaceae’s decreased abundance. Normalized abundances of several genera, increasing *Lachnospiraceae_NK4A136_group*, etc., and decreasing *Eubacterium_nodatum_group* and *Staphylococcus*	Serum creatinine and BUN levels decreased markedly, and the creatinine clearance rate rose. Preserved residual kidney function and alleviated kidney injury, shown by reduced glomerular size, lower glomerulosclerosis index, and less kidney interstitial fibrosis.	[102]
Puerariae lobatae formulae	Adenine- and potassium oxonate-induced hyperuricemia model mice	*n* = 6	16S rRNA sequencing	Radix Puerariae Lobatae, Folium Mori, Fructus Chaenomelis, Poria, Semen Coicis, Fructus Litchi, Herba Taraxaci, Fructus Gardeniae, Rhizoma Dioscoreae, Radix et Rhizoma Glycyrrhizae, Radix Ginseng, Cortex Cinnamomi, and Cordyceps Militaris fruiting bodies	Restored microbial diversity and altered the gut microbiota structure; enriched the abundance of the beneficial bacterium *Akkermansia* at both the genus and species levels	Inhibited XOD and ADA to reduce uric acid, and lowered IL-6 and IL-1β, inhibiting inflammation. Reduced plasma CREA, UREA, ALT, and AST. Eased liver (edema, swelling) and kidney (tubular expansion, atrophy) problems	[103]
Huang Gan formula	Adenine-induced CKD rats	*n* = 7–10	16S rRNA sequencing	Rhubarb (Dahuang) and licorice root (Gancao)	Modulated gut microbiota in CKD. Restored microbial community structure via normalized beta diversity and Firmicutes/Bacteroidetes ratio. Enriched SCFA-producing genera like *Prevotella*, *Bacteroides*, and *Coprococcus* and suppressed conditional pathogens such as Clostridia and *Ruminococcus*	Improved kidney function (lowered SCR, BUN, UA, T-CHO). Eased kidney pathology (curbed matrix expansion, etc.). Halted inflammation (downregulated pro-inflammatory cytokines, upregulated IL-10, blocked NF-κB). Reduced gut-derived uremic toxins.	[104]
Yishen Qingli Heluo Granule	5/6 nephrectomy CKD rats	*n* = 6	16S rRNA sequencing	Angelicae Sinensis Radix (Danggui, DG), Achyranthis Bidentatae Radix (Niuxi, NX), Centella Asiatica (L.) Urban (Jixuecao, JXC), Polygonati Rhizoma (Huangjing, HJ),Smilacis Glabrae Rhixoma (Tufuling, TFL), Radix Rhei Et Rhizome (Dahuang, DH), Pyrrosiae Folium (Shiwei, SW), Hedysarum Multijugum Maxim (Huangqi, HQ), Serissa Japonica (Thunb.) Thunb (Liuyuexue, LYX), Polygoni Cuspidati Rhizoma Et Radix (Huzhang, HZ)	Gut microbiota was reshaped, which was characterized by a reduced ratio of Firmicutes/Bacteroidota	Improved kidney function and fibrosis in 5/6 nephrectomized rats. Decreased expression of PTGS2 and IL-6, and increased expression of p53 in kidney tissue	[105]
Xiaoyu Xiezhuo Decoction	Unilateral ureteral obstruction (UUO) rats	*n* = 6	16S rDNA sequencing	Rhubarb, Peach Kernel, Achyranthes, Earthworm, and Astragalus	Modulated gut microbiota. Restored structure with beta-diversity shift, normalized ratio, boosted SCFA-producers (Allobaculum, etc.), increasing butyrate, and reduced Aerococcus	Eased kidney injury and fibrosis in UUO—CKD. Inhibited glomerular and tubular damage, mesangial and matrix expansion, inflammation, and fibrosis markers. Medium dose as effective as Irbesartan	[106]

Abbreviations: GM, gut microbiota; CKD, chronic kidney disease; U-ALB, urinary albumin; TMAO, trimethylamine N-oxide; UUO, unilateral ureteral obstruction; Nx, nephrectomy; XOD, xanthine oxidase; ADA, adenosine deaminase; IL, interleukin; CREA, creatinine; ALT, alanine aminotransferase; AST, aspartate aminotransferase; SCFA, short-chain fatty acids; SCR, serum creatinine; BUN, blood urea nitrogen; UA, uric acid; T-CHO, total cholesterol; NF-κB, nuclear factor kappa-B; PTGS2, prostaglandin-endoperoxide synthase 2.

### 3.2. Single Herbs

Intervention studies with single herbs have also elucidated clear pathways for their efficacy via the gut microbiota in CKD (Table 2). A rhubarb enema increased the abundance of symbiotic and probiotic bacteria while reducing potential pathogens in CKD rats, consequently decreasing serum TMAO levels and improving kidney inflammation and fibrosis [107]. *Puerariae lobatae* Radix rescued gut microbiota dysbiosis in a salt-loaded murine model, significantly enriching *Akkermansia* and *Bifidobacterium*, whose abundances were positively correlated with the maintenance of normal kidney histology and function, thereby alleviating kidney fibrosis [108].

*Astragalus* modulated the gut microbiota structure in a hyperuricemic nephropathy rat model by reducing the F/B ratio, upregulating *lactobacilli*, and enriching specific biomarkers like *Faecalibaculum*. This led to kidney-protective effects, manifested as decreased serum uric acid, creatinine, and BUN levels, along with attenuated kidney inflammation and histological damage [109]. Furthermore, Paotianxiong and Panax notoginseng saponins were shown to restore gut microbial alpha diversity, increase beneficial bacteria like *Lactobacillus*, and ameliorate kidney injury through mechanisms involving the modulation of neuroendocrine-immune balance and inhibition of the NLRP3 inflammasome. Relevant studies are summarized in Table 2.

**Table 2:** Basic studies on the potential mechanisms of single herbs in ameliorating CKD.

Single Herb	Subject	Subject Size	Gut Microbiota Study Method	Effect on GM	Relief or Treatment of Symptoms	Reference
Rhubarb	5/6 nephrectomy CKD rats	*n* = 10	16S rRNA sequencing	Increased the abundance of some symbiotic bacteria and probiotics, while reducing the abundance of some potential pathogens at the genus level	Reduced serum TMAO and trimethylamine (TMA) levels, inhibited the expression of inflammatory markers (interleukin-6, tumor necrosis factor α, and interferon-γ), and alleviated tubular atrophy, monocyte infiltration, and interstitial fibrosis	[106]
Danshen	Adenine-induced CKD rats	*n* = 6	16S rRNA sequencing	Regulated *Shuttleworthia*, *Pseudomona*, *Peptococcus*, *Ruminococcus*, and *Peptostreptococcaceae*	Alleviated key symptoms of chronic kidney failure in rats, including glomerular and tubular atrophy, urate deposition, and interstitial fibrosis	[110]
Puerariae lobatae Radix	2% NaCl-feeding murine model	*n* = 6	16S rDNA sequencing	Rescued the gut microbiota dysbiosis and enriched *Akkermansia* and Bifidobacterium, the relative abundances of which were positively correlated with normal maintenance of kidney histology and function	Alleviated CKD-associated increases in creatinine and urine protein and nephritic histopathological injury. Protected kidney from fibrosis	[108]
Astragalus	Potassium oxonate and adenine induced hyperuricemic rat model	*n* = 6	16S rRNA sequencing	Demonstrated modulation of gut microbiota structure by increasing alpha-diversity indices, altering beta-diversity patterns, reducing the Firmicutes/Bacteroidetes ratio, upregulating beneficial *lactobacilli*, and enriching specific biomarkers including *Faecalibaculum* and *Clostridiaceae*, thereby ameliorating hyperuricemia-related dysbiosis	Demonstrated kidney-protective effects by reducing liver and kidney indices, improving kidney function (lowering UA, Scr, BUN), alleviating inflammation (IL-6, IL-1β), and attenuating histological damage like fibrosis and collagen deposition	[109]
Paotianxiong	Adenine-induced CKD rats	*n* = 6	Metagenome sequencing	Eased CKD by fixing intestinal dysbiosis: restored diversity, boosted good bacteria, adjusted *Prevotella*, and reversed imbalances (fecal microbiota transplantation validated)	Ameliorated chronic kidney disease (CKD) via multiple mechanisms: reduced kidney pathological damage and kidney index; modulated neuroendocrine–immune regulation (lowered ACTH/cGMP, elevated cAMP); suppressed systemic inflammation (reduced IL-1β, IL-6, TNF-α); improved intestinal pathology, with fecal transplantation confirming gut–kidney axis mediation	[111]
Panax notoginseng saponins	Adenine-induced CKD rats	*n* = 7	16S rRNA sequencing	Demonstrated modulatory effects on gut microbiota diversity in CKD, restoring differential microbiota at both phylum and genus levels. Re-established the microbial barrier and regulated bacterial metabolites, potentially contributing to CKD amelioration	Boosted kidney function (raised key indicators, lowered excess ones). Improved kidney histology. Reduced systemic inflammation. Inhibited kidney activation, eased fibrosis, and reduced kidney cytokines	[112]

Abbreviations: GM, gut microbiota; CKD, chronic kidney disease; TMA, trimethylamine; TMAO, trimethylamine N-oxide; IL, interleukin; TNF-α, tumor necrosis factor-alpha; UA, uric acid; Scr, serum creatinine; BUN, blood urea nitrogen; ACTH, adrenocorticotropic hormone; cGMP, cyclic guanosine monophosphate; cAMP, cyclic adenosine monophosphate.

### 3.3. Herbal Ingredients

Active herbal ingredients, with their defined chemical structures, allow for more in-depth mechanistic research (Table 3). Berberine increased the abundance of Bacteroidota and reduced the F/B ratio in adenine-induced CKD rats. It inhibited the production of gut-derived uremic toxins, particularly p-cresol sulfate, and lowered serum levels of pro-inflammatory cytokines (TNF-α, IL-6, IL-1β), thereby preserving glomerular structure [113]. Puerarin, a primary active component of *Puerariae lobatae* Radix, increased microbial alpha-diversity, significantly enriched *Akkermansia*, and reduced pathogenic bacteria like *Bacteroides* and *Proteobacteria*. It lowered uric acid levels by suppressing xanthine oxidase activity and reducing inflammatory cytokines, ultimately improving kidney function [103].

Other ingredients, such as Alisol B 23-acetate, reversed the aberrant abundance of 10 genera in 5/6 nephrectomized rats, subsequently lowering blood pressure, reducing proteinuria, and inhibiting kidney fibrosis [114]. Isoquercitrin directly reduced the production of the uremic toxin IS by regulating the bacterial electron transport chain to inhibit tryptophan transport and indole biosynthesis [115]. Curcumin protected the intestinal barrier, increased SCFA-producing bacteria, improved metabolic endotoxemia, and enhanced kidney function [116]. Relevant studies are summarized in Table 3.

## 4. Discussion

The role and mechanisms of the gut microbiota in the development and progression of CKD have been extensively studied and partially elucidated. Current research primarily focuses on the impact of gut microbiota-derived uremic toxins (such as TMAO, IS, and pCS) and intestinal barrier dysfunction on CKD progression. Among the TCM interventions summarized in this review, certain formulae, single herbs, and active ingredients stand out as the most promising for alleviating CKD progression and improving renal function:

(1) Herbal formulae: *Suyin Detoxification Granule* and the combination of *Astragalus membranaceus-Salvia miltiorrhiza*—the former reverses gut Firmicutes/Bacteroidota dysbiosis, suppresses pathogenic *Muribaculaceae*, and inhibits TMAO-induced kidney tubular ferroptosis to block fibrosis [98]; the latter normalizes the Firmicutes/Bacteroidetes ratio, and enriches butyrate-producing *Akkermansia* and lactate-producing *Lactobacillus*, thereby reducing uremic toxins (e.g., pCS, IS) and improving oxidative stress [97].
(2) Single herbs: *Rhubarb* and *Puerariae lobatae Radix*—Rhubarb enema increases symbiotic bacteria abundance, lowers serum TMAO, and alleviates kidney interstitial fibrosis [107]; *Puerariae lobatae Radix* enriches *Akkermansia* and *Bifidobacterium* to preserve kidney histology and reduce proteinuria, via downregulating the Wnt/β-catenin pathway [108].
(3) Active ingredients: Berberine and Curcumin—Berberine increases gut Bacteroidota abundance, inhibits the tyrosine–p-cresol–pCS pathway to reduce uremic toxin production, and lowers pro-inflammatory cytokines (TNF-α, IL-6) [113]; curcumin protects the intestinal barrier by suppressing opportunistic pathogens (e.g., *Escherichia-Shigella*), enriches SCFA-producing *Lactobacillus*, and ameliorates metabolic endotoxemia to preserve kidney function [116].

However, current research on TCM interventions remains predominantly descriptive. While numerous studies demonstrate that TCM can remodel the microbiota composition and improve kidney phenotypes, most evidence is derived from preclinical animal models, and causal relationships often remain established only at the associative level. A significant gap exists in moving from “correlation” to “causation”. To bridge this gap and synthesize a robust mechanistic understanding, future research must adopt a hierarchical strategy. First, phenotypic validation using classic CKD models (e.g., adenine-induced, 5/6 Nx) remains the foundation. Second, to move beyond simple abundance profiling, untargeted metabolomics combined with metagenomics should be employed to identify specific bacterial strains responsible for uremic toxin synthesis, rather than just phylum-level changes. Crucially, causality must be established through rigorous intervention studies, such as fecal microbiota transplantation (FMT) or mono-colonization with specific strains identified in TCM studies, to determine whether the microbiota alone can reproduce the therapeutic effects of TCM. Finally, integrating host-side validation via single-cell transcriptomics or proteomics in kidney tissues is essential to map the downstream signaling pathways (e.g., NF-κB, TGF-β/Smad) modulated by these microbial shifts. This research framework enables mechanistic studies on TCM regulation of the “gut–kidney axis” at the single microbe and single metabolite levels.

In addition to the aforementioned microbial mechanisms, research on the active ingredients of TCM requires more rigorous pharmacokinetic and bioavailability studies to identify bioactive monomer compounds that can enter systemic circulation. These studies are critical not only for clarifying the effective dose and target tissue distribution of TCM ingredients, but also for evaluating their potential toxic effects—for example, whether high concentrations of certain components (e.g., berberine, curcumin at supra-therapeutic doses) induce hepatotoxicity, nephrotoxicity, or intestinal microbiota dysbiosis (a paradoxical risk given their role in modulating gut microbiota). Furthermore, how TCM reduces uremic toxins, repairs the intestinal barrier, and regulates intestinal immunity as upstream mechanisms for its kidney-protective effects warrants in-depth investigation. Future work should prioritize elucidating the direct interactions between active TCM components and the gut microbiota, and how these interactions ultimately regulate the production of uremic toxins and the integrity of the intestinal barrier, thereby identifying new intervention targets for CKD treatment.

Despite the promising therapeutic potential, several critical translational challenges must be addressed to avoid an overly optimistic outlook. (1) Standardization: The complexity of TCM formulae leads to batch-to-batch variability. Future studies must utilize rigorous quality control to ensure reproducibility. (2) Safety: The potential nephrotoxicity of certain herbal components cannot be ignored. Detailed pharmacokinetic studies are required to evaluate the safety profile of TCM-derived metabolites in the context of impaired kidney function. (3) Complexity of Interactions: Unlike single-target drugs, TCM acts on a network. Recent studies utilizing multi-omics and network pharmacology have begun to decode this complexity, but standardized protocols for these analyses are still needed. (4) Clinical Evidence Gap: A notable disparity exists between the extensive historical clinical application of TCM and the scarcity of modern research specifically linking its efficacy to gut microbiota modulation in humans. As reflected in our review, the majority of mechanistic data is derived from animal models. This reliance on preclinical data is largely due to the inherent difficulties in controlling confounding factors (e.g., diet, lifestyle, genetics) that significantly influence the human microbiome, making it challenging to isolate the specific effects of TCM. Consequently, while animal studies provide essential proof-of-concept, future research must prioritize high-quality randomized controlled trials (RCTs) with microbiome-specific endpoints to validate whether the gut-modulating effects observed in rodents are reproducible in human CKD patients.

In summary, while TCM offers a rich resource for microbiota-targeted therapies, the field must transition from descriptive studies to mechanistic dissection and rigorous clinical validation.

## 5. Conclusions

The gut microbiota is a key player in CKD pathogenesis via the “gut–kidney axis”, where dysbiosis increases detrimental uremic toxins (e.g., TMAO, IS, pCS) and reduces beneficial metabolites (e.g., SCFAs), driving inflammation and fibrosis.

Collectively, these TCM interventions confirm that TCM—including formulae, single herbs, and active compounds—offers a promising therapeutic strategy by targeting the gut–kidney axis. Evidence indicates that TCM achieves renoprotective effects primarily by remodeling gut microbiota homeostasis, decreasing uremic toxin production (e.g., TMAO, pCS), enhancing beneficial metabolites (e.g., SCFAs), and restoring gut barrier integrity—mechanisms that directly counteract the core pathological drivers of CKD. Strategically manipulating the gut microbiota with TCM is thus a promising frontier for CKD therapy. However, further mechanistic studies are crucial to precisely define the interactions between specific TCM components, their microbial targets, and host responses, to optimize future clinical applications.

Current findings consistently demonstrate that TCM can reduce toxin production and restore barrier integrity in animal models. However, clinical evidence remains preliminary. To translate these findings into clinical practice, future research must focus on (1) validating causal mechanisms using specific microbial metabolites and colonized models; (2) establishing standardized protocols for TCM preparation and safety evaluation; and (3) conducting large-scale, multi-center clinical trials to confirm the efficacy and safety of TCM interventions in diverse CKD populations.

## Figures and Tables

**Table 3:** Basic studies on the potential mechanism of herbal ingredients against CKD.

Ingredient	Subject	Subject Size	Gut Microbiota Study Method	Effect on GM	Relief or Treatment of Symptoms	Reference
Madecassoside	Unilateral ureteral obstruction (UUO) mice	*n* = 6	16S rDNA sequencing and qPCR assay	Promoted *Bacteroides fragilis* growth	Ameliorated kidney fibrosis; improved kidney morphology and the kidney index	[14]
Berberine	Adenine-induced CKD rats	*n* = 10	16S rRNA sequencing	Increased the abundance of bacteria in the phylum Bacteroidota and reduced Firmicutes/Bacteroidota ratio	Lowered serum creatinine and BUN; preserved glomerular structure and reduced tissue damage; decreased pro-inflammatory cytokines (TNF-α, IL-6, IL-1β) by >30% vs. the model group; reduced uremic toxins (especially those in the tyrosine–p-cresol–p-cresol sulfate pathway), thereby lowering plasma p-cresol sulfate concentration	[113]
Alisol B 23-acetate	5/6 nephrectomized and unilateral ureteral obstructed rats	*n* = 7	16S rRNA sequencing	Reversed the aberrant abundance of 10 out of the 12 genera that were significantly altered in 5/6 nephrectomized rats	Lowered blood pressure, reduced serum creatinine and proteinuria, suppressed expression of RAS constituents, and inhibited the epithelial-to-mesenchymal transition and Smad7-mediated inhibition of Smad3 phosphorylation	[114]
isoquercitrin	Adenine-induced CKD model mice	*n* = 6	16S rDNA sequencing	Regulated the gut bacterial electron transport chain to inhibit the establishment of H proton potential, thereby inhibiting the transport of tryptophan and further reducing indole biosynthesis	Reduced plasma IS concentration in CKD model mice. Did not improve kidney function or IS clearance, nor reduce circulating IS in mice with normal kidney function	[115]
Curcumin	Adenine- and potassium oxonate-induced CKD rats	*n* = 10	16S rRNA sequencing	Protected against the overgrowth of opportunistic pathogens in UAN, including *Escherichia-Shigella* and *Bacteroides*, and increased the relative abundance of bacteria producing SCFAs, such as *Lactobacillus* and *Ruminococcaceae*	Decreased serum uric acid, serum creatinine, and BUN level. Attenuated kidney pathological lesions and metabolic endotoxemia, and improved tightly linked protein expression	[116]
Puerarin	Adenine- and potassium oxonate-induced hyperuricemia model mice	*n* = 6	16S rRNA sequencing	Increased microbial alpha-diversity and induced distinct changes in the overall microbial community structure. Reduced the relative abundance of pathogenic bacteria, including Bacteroides and Proteobacteria, while markedly enriching beneficial bacteria, most notably *Akkermansia*, across the family, genus, and species levels.	Lowered uric acid levels by suppressing XOD and ADA activity and reduced the expression of inflammatory markers IL-6 and IL-1β. Led to a significant reduction in plasma levels of CREA, UREA, ALT, and AST. Ameliorated extensive edema, hepatocyte swelling, cytoplasmic thinning, and vacuolization in the liver, along with severe tubular expansion, vacuolar degeneration, glomerular atrophy, and mild inflammatory infiltration in the kidneys	[103]

Abbreviations: GM, gut microbiota; UUO, unilateral ureteral obstruction; CKD, chronic kidney disease; TNF-α, tumor necrosis factor-alpha; IL, interleukin; RAS, renin–angiotensin system; UAN, uric acid nephropathy; SCFAs, short-chain fatty acids; XOD, xanthine oxidase; ADA, adenosine deaminase; CREA, creatinine; ALT, alanine aminotransferase; AST, aspartate aminotransferase.

## Data Availability

No new data were created or analyzed in this study.
